# Pollution Characteristics and Source Apportionment of Black Carbon Aerosols during Spring in Beijing

**DOI:** 10.3390/toxics12030202

**Published:** 2024-03-05

**Authors:** Wenkai Lei, Xingru Li, Zhongyi Yin, Lan Zhang, Wenji Zhao

**Affiliations:** 1 College of Resource Environment and Tourism, Capital Normal University, Beijing 100048, China; 18601232683@163.com (W.L.);; 2 Department of Chemistry, Capital Normal University, Beijing 100048, China

**Keywords:** black carbon, optical characteristics, concentration characteristics, linear regression, potential source area

## Abstract

Black carbon (BC) aerosols are important for absorbing aerosols, affecting global climate change and regional air quality, and potentially harming human health. From March to May 2023, we investigated black carbon aerosol levels and air pollution in Beijing. Employing methods such as linear regression, Potential Source Contribution Function (PSCF) and Concentration-Weighted Trajectory (CWT), we analyzed the characteristics and sources of black carbon aerosols in the region. Results indicate that the light absorption coefficients of BC and BrC decrease with increasing wavelength, with BrC accounting for less than 40% at 370 nm. Daily variations in BC and PM_2.5_ concentrations exhibit similar trends, peaking in March, and BC displays a distinct bimodal hourly concentration structure during this period. Aethalometer model results suggest that liquid fuel combustion contributes significantly to black carbon (1.08 ± 0.71 μg·m^−3^), surpassing the contribution from solid fuel combustion (0.31 ± 0.2 μg·m^−3^). Furthermore, the significant positive correlation between BC and CO suggests that BC emissions in Beijing predominantly result from liquid fuel combustion. Potential source area analysis indicates that air masses of spring in Beijing mainly originate from the northwest (40.93%), while potential source areas for BC are predominantly distributed in the Beijing–Tianjin–Hebei region, as well as parts of the Shandong, Shanxi and Henan provinces. Moreover, this study reveals that dust processes during spring in Beijing have a limited impact on black carbon concentrations. This study’s findings support controlling pollution in Beijing and improving regional air quality.

## 1. Introduction

In recent years, widespread atmospheric aerosol pollution characterized by significant spatial variation and complex chemical composition has emerged as a pressing global concern. This issue affects both China and the world at large [1], influencing global and regional climate dynamics and posing risks to public health [2]. Consequently, aerosols have garnered substantial attention within the field of atmospheric science. Among these aerosols, BC is a dominant anthropogenic source and plays a pivotal role in shaping the global atmospheric climate [3].

Black carbon aerosols primarily originate from the incomplete combustion of carbon-containing fossil fuels and biomass, which can be classified into natural and anthropogenic sources. While natural events such as volcanic eruptions and forest fires constitute significant natural sources of black carbon emissions, their sporadic occurrences contribute minimally to long-term background concentrations of black carbon in the atmosphere [4]. In contrast, anthropogenic sources release black carbon on a broader and more persistent scale. BC, often a component of PM_2.5_, possesses chemical inertness, typically exists at sub-micron sizes, and has an extended atmospheric lifespan. Thus, BC serves as a valuable indicator of human impact on the atmosphere and facilitates the tracking of polluted air masses originating from diverse source regions. Moreover, due to its small particle size and suspension capabilities, BC can directly infiltrate human respiratory tracts and alveoli, leading to a range of health issues and posing a significant threat to public well-being [5,6,7].

While atmospheric BC concentrations are generally low and exhibit uneven global distribution, BC exerts a potent solar radiation absorption effect across the visible and infrared spectra. By absorbing and scattering light at varying wavelengths, black carbon alters local atmospheric stability and vertical structures, thereby influencing regional weather patterns and global atmospheric circulation [8]. Current research, both domestic and international, has predominantly explored temporal variations in BC concentration, key influencing factors, source analyses, as well as the radiative forcing and climatic implications of BC aerosols. Analyzing BC concentrations across different locations in Mexico, Peralta et al. [9] found a strong correlation between BC and CO concentrations in urban areas, with the correlation being more pronounced than in suburban and high-altitude regions. Similarly, Barman and Gokhale [10] used BC data obtained from 2016 to 2017 in Guwahati, India, to study the deposition of BC concentration under different meteorological conditions, and applied the mixed single-particle Lagrangian integral trajectory (HYSPLIT) model to analyze the area and source distribution of BC possible deposition. Furthermore, Li et al. [11] conducted continuous BC and BrC measurements during the heating season in Beijing. They employed trajectory calculations via the HYSPLIT model and employed the Concentration-Weighted Trajectory (CWT) model, as well as the MODIS fire point map, to analyze potential pollution sources. Several studies have highlighted the role of black carbon aerosols in impeding the development of the atmospheric boundary layer by altering surface radiation energy balances and heating the boundary layer top, thereby exacerbating severe haze pollution in urban centers [12,13,14]. Given its status as one of the world’s major black carbon emission centers [15,16], China plays a pivotal role in shaping global atmospheric conditions and climate dynamics. Beijing, as China’s political, cultural and technological epicenter and the core city of the Beijing–Tianjin–Hebei economic zone, is characterized by extensive industrial development, a substantial population and a high volume of motor vehicles. The substantial consumption of fossil fuels has led to increased BC aerosol emissions [17,18]. Moreover, Beijing’s warm temperate monsoon climate, characterized by minimal spring rainfall and dry, windy conditions, exacerbates the occurrence of pollution-related weather events [19]. Therefore, a comprehensive analysis of BC aerosol pollution characteristics and potential sources in the atmosphere of Beijing’s urban area during spring holds profound significance for improving regional atmospheric conditions.

In this study, we employed observational data, encompassing BC concentrations, to scrutinize the optical characteristics of aerosols and the concentration patterns of BC during the spring season in Beijing’s urban area. We conducted linear regression analyses correlating BC concentrations with atmospheric pollutants (NO_2_, CO, O_3_). Subsequently, we applied the Potential Source Contribution Function (PSCF) and CWT methods to delineate the potential source regions of BC and assess whether dust weather exerts an influence on BC concentrations in Beijing.

## 2. Materials and Methods

### 2.1. Study Region and Data Collection

**Figure 1:**
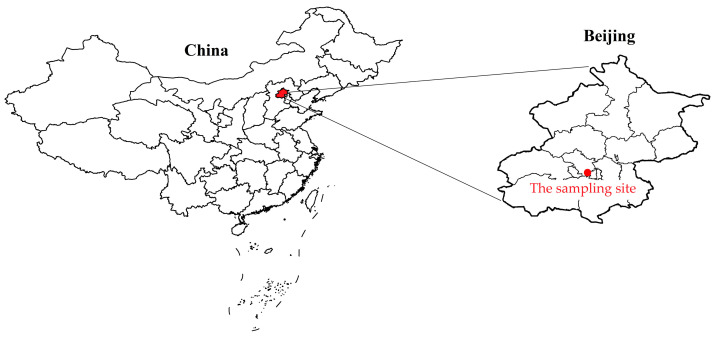
Location of the sampling site in this study.

The instrument was positioned on the third-floor platform of the experimental building at Beijing Capital Normal University (39.9302° W, 116.3034° E). It is situated at an elevation of approximately 10 m above ground level. The sampling site (Figure 1) is surrounded by residential areas and schools, with its eastern side in close proximity to the main road. To a considerable extent, the data collected from this monitoring site serves as a representation of the atmospheric black carbon aerosol pollution levels within Beijing’s urban area. In this research, a black carbon meter (AE33, Magee Scientific, Berkeley, CA, USA) was employed to measure the light absorption and the corresponding aerosol absorption concentration at seven distinct wavelengths (370, 470, 520, 590, 660, 880 and 950 nm). About the instrument, it adopts two parallel point measurement technology and dynamic zero-point calibration, which provides almost real-time compensation for point loading effect. The detection limit is less than 0.005 μg·m^−3^ per hour, the detection accuracy of 0.03 μg·m^−3^ per minute and the concentration resolution of 0.001 μg·m^−3^. The measurements were conducted at a constant flow rate of 5 L/min and a temporal resolution of 1 min. Given that the absorption effect of BC dominates and the absorption efficiency of other aerosols can be disregarded at the 880 nm wavelength, the BC concentration was determined by monitoring the attenuation of the aerosol beam as it passed through a quartz fiber filter at 880 nm. Data collection occurred during the spring season of 2023, spanning from March 1 to May 22. After eliminating data recorded during special meteorological conditions, power outages and other factors, a total of 78,840 min of valid data was retained for analysis. The daily and monthly mean values of BC, PM_2.5_ and PM_10_ are calculated from the hourly mean data. Meteorological parameters such as temperature (°C), wind speed (m/s) and wind direction were measured using the automatic weather observation equipment (CAWS600, HuaYun Group, Beijing, China) situated at the campus weather station. Concentrations of air pollutants (PM_10_, PM_2.5_, NO_2_, O_3_, CO) were sourced from the automatic observation instrument (MW600, HuaYun Group, China) set up near the campus automatic weather station, and the time resolution is 1 h. Of these, the error range of PM_2.5_ and PM_10_ is less than 10%, and the error range of CO, O_3_ and NO_2_ is less than 3%. These instruments are only 300 m away from the sampling site.

### 2.2. Black Carbon Measurements

The formula for the absorption coefficient (Abs) of carbonaceous aerosols at specific wavelengths is as follows:

(1) $$Abs\left(\lambda \right)=\sigma_{air}\times M_{BC\left(\lambda \right)}\times 10^{-3}$$

In the formula, $\sigma_{air}$ represents the mass absorption cross-section. Measured $\sigma_{air}$ values at seven different wavelengths (370, 470, 520, 590, 660, 880 and 950 nm) are as follows: 18.47, 14.54, 13.14, 11.58, 10.35, 7.77 and 7.19 m^2^·g^−1^, respectively. $M_{BC(\lambda )}$ denotes the black carbon mass concentration specific to a given wavelength, ng·m^−2^.

Assuming negligible light absorption by dust, Abs(*λ*) can be separated into BC absorption and BrC absorption. Given the dominant absorption effect of BC at 880 nm and the negligible absorption efficiency of other aerosols, ${Abs}_{BC}\left(\lambda \right)$ and ${Abs}_{BrC}\left(\lambda \right)$ at other wavelengths can be estimated using the following formulas.

(2) $$Abs\left(\lambda \right)={Abs}_{BC}(\lambda )+{Abs}_{BrC}(\lambda )$$

(3) $${Abs}_{BC}\left(\lambda \right)=Abs\left(880\right)\times {\left(\frac{\lambda }{880}\right)}^{{-AAE}_{BC}}\;\;$$

(4) $${Abs}_{BrC}\left(\lambda \right)=Abs\left(\lambda \right)-Abs\left(880\right)\times {\left(\frac{\lambda }{880}\right)}^{{-AAE}_{BC}}\;\;\;$$

${Abs}_{BC}\left(\lambda \right)$ signifies the light absorption attributable to BC at a particular wavelength λ, while ${Abs}_{BrC}\left(\lambda \right)$ represents the light absorption contribution of BrC at the same wavelength. AAEBC refers to the Ångström index, which characterizes the spectral dependence of BC. In this study, a value of 1.1 was employed based on previous research [11].

Some scholars [20] contend that the incomplete combustion of liquid fuels (e.g., traffic emissions) and solid fuels (e.g., coal and biomass) are the two primary sources of black carbon aerosols in the atmosphere. The total absorption coefficient of BC at a specific wavelength can be expressed as follows:

(5) $$Abs\left(\lambda \right)=Abs{\left(\lambda \right)}_{liquid}+Abs{\left(\lambda \right)}_{solid}$$

In this equation, $Abs{\left(\lambda \right)}_{liquid}$ and $Abs{\left(\lambda \right)}_{solid}$ represent the black carbon absorption coefficient resulting from liquid fuel and solid fuel emissions, respectively.

(6) $$\frac{Abs{\left(370\;nm\right)}_{liquid}}{Abs{\left(880\;nm\right)}_{liquid}}=({\frac{370}{880})}^{{-AAE}_{liquid}}$$

(7) $$\frac{Abs{\left(370\;nm\right)}_{solid}}{Abs{\left(880\;nm\right)}_{solid}}=({\frac{370}{880})}^{{-AAE}_{solid}}$$

(8) $${BC}_{liquid}=M_{BC}(880\;nm)\frac{Abs{\left(880\;nm\right)}_{liquid}}{Abs(880\;nm)}$$

(9) $$BC={BC}_{liquid}+{BC}_{solid}$$

Additionally, ${AAE}_{liquid}$ and ${AAE}_{solid}$ are the wavelength exponents of the BC absorption coefficients associated with liquid fuel and solid fuel. Based on the findings of Jing et al. [18], this study adopts the assumptions ${AAE}_{liquid}$ = 1.0 and ${AAE}_{solid}$ = 2.2. Consequently, the mass concentration of BC generated by liquid fuel and solid fuel emissions can be calculated using the above formula.

### 2.3. PSCF and CWT Models

CWT model is a method used to compute the potential pollution source regions for air pollutants and assess the degree of pollution along specific trajectories [21]. This approach involves the calculation of weighted concentrations for each grid point, taking into account the trajectories passing through the grid and the corresponding black carbon (BC) concentrations associated with each trajectory.

(10) $$C_{ij}=\frac{1}{\sum_{l=1}^{N}n_{ijl}}\sum_{l=1}^{N}C_{l}n_{ijl}$$

$C_{ij}$ represents the average weighted concentration at grid point (*i, j*). N stands for the total number of trajectories passing through the grid (*i*, *j*), while $C_{l}$ denotes the BC mass concentration linked to the trajectory labeled as “*l*” that passes through the same grid and $n_{ijl}$ represents the residence time associated with the ‘*l*-th’ trajectory as it passes through the grid.

PSCF model is computed based on a spatial grid and is defined as the ratio of the number of endpoints (*mij*) of pollution air trajectories passing through a specific grid (*ij*) in the study area (when the feature values exceed a set pollution threshold) to the total number of endpoints (*nij*) passing through that grid.

(11) $${PSCF}_{ij}=\frac{m_{ij}}{n_{ij}}$$

As PSCF is a conditional probability function, the uncertainty of PSCF values increases when the airflow residence time within each grid is short (indicating smaller *mij* values). Therefore, it is necessary to introduce an empirical weighting function, *W*(*nij*), for interval weighting and error reduction. The weight function, *W*(*nij*), is defined as follows:

(12) $$W_{ij}=\left\{\begin{array}{c} 1.0,\;{\;\;\;\;n}_{ij}>120\\ 0.8,\;\;120\geq n_{ij}>40\\ 0.4,\;\;{40\geq n}_{ij}>20\;\\ 0.2,\;\;n_{ij}\geq 20 \end{array}\right.$$

Consequently, weighted calculations can be applied to PSCF:

(13) $${WPSCF}_{ij}={PSCF}_{ij}\times W_{ij}$$

During CWT and PSCF analyses, air trajectories are divided into a 0.2° × 0.2° grid. Different colors represent the size of the weighted concentrations and weighted contribution functions within each grid cell. The results of both methods are visualized. PSCF and CWT analyses were calculated using Meteinfo Map 3.7.0 software. Meteorological parameters used by the software are sourced from NOAA’s website (ftp://arlftp.arlhq.noaa.gov/pub/archives/gdas1 (accessed on 20 October 2023)), and fire point data are obtained from NASA’s website (FIRMS—Creating Archive Download Request (nasa.gov) (accessed on 21 October 2023)).

## 3. Results and Discussion

### 3.1. Light Absorption Characteristics

**Figure 2:**
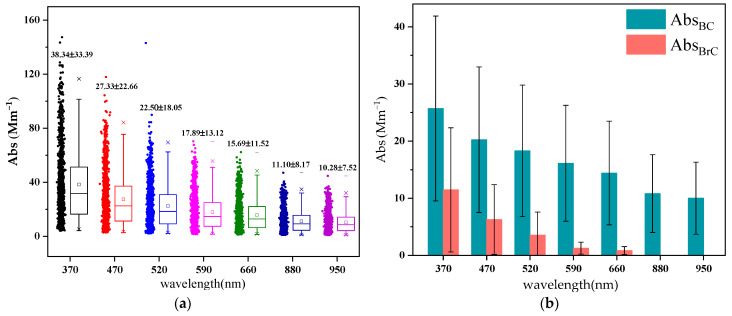
(**a**) Box-and-whisker plot of absorption coefficients at seven wavelengths as measured with AE33. (**b**) Average values of Abs_BC_ and Abs_BrC_ at different wavelengths.

Figure 2a presents a box plot of the aerosol optical absorption (Abs) coefficient within the wavelength range of 370–950 nm. It is evident that the Abs value is highest at 370 nm and decreases as the wavelength increases. This trend has been confirmed in numerous previous studies [11,22]. The average absorption coefficients for the seven wavelengths were as follows: 38.34 ± 33.39 Mm^−1^ (370 nm), 27.33 ± 22.66 Mm^−1^ (470 nm), 22.51 ± 8.05 Mm^−1^ (520 nm), 17.89 ± 13.12 Mm^−1^ (590 nm), 15.69 ± 11.52 Mm^−1^ (660 nm), 11.1 ± 8.17 Mm^−1^ (880 nm) and 10.28 ± 7.52 Mm^−1^ (950 nm), respectively. Figure 2b displays Abs_BC_ and Abs_BrC_ at various wavelengths, showing a consistent gradual and steady decrease from ultraviolet to visible light, with Abs_BrC_ being virtually non-existent at 880 and 950 nm. Consequently, previous studies have generally considered Abs_BC_ at 880 nm as pure BC absorption, while Abs_BrC_ at 370 nm represents BrC absorption [23,24].

**Figure 3:**
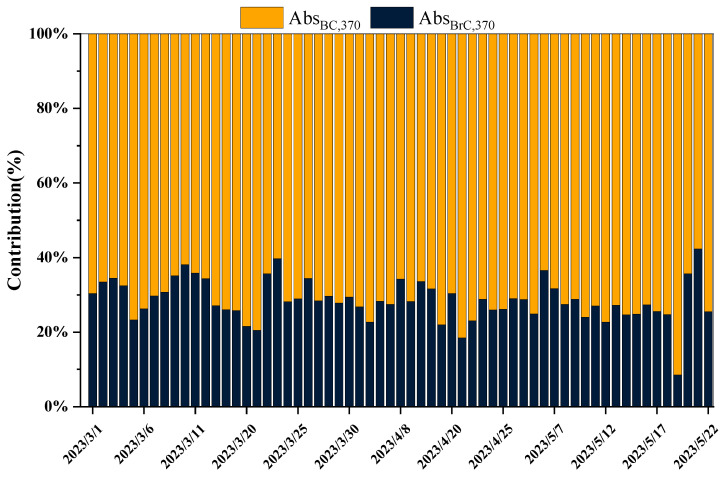
The absorption contribution of Abs_BC_ and Abs_BrC_ at 370 nm.

The Abs_BC_ at 880 nm and Abs_BrC_ at 370 nm in spring were 10.82 ± 6.8 Mm^−1^ and 11.47 ± 10.87 Mm^−1^, respectively. Figure 3 illustrates the daily average proportion of BrC and BC at 370 nm. Throughout the study period, the proportion of Abs_BrC_, 370 nm was consistently less than 40%, with only a few exceptional days exceeding this threshold. The average proportion during spring was 28.57% ± 5.47%, a result consistent with the spring online monitoring findings in Lanzhou [22]. Additionally, changes in aerosol sources, such as shifts in solid fuel combustion, may contribute to variations in Abs values [25,26].

### 3.2. BC Concentration Characteristics

**Figure 4:**
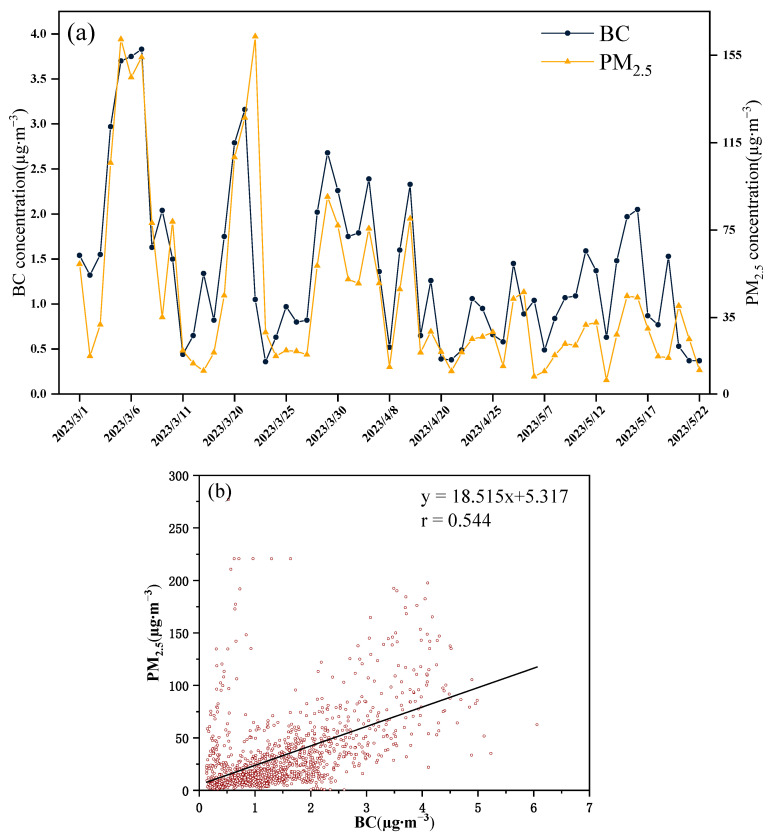
(**a**) Variation of BC and PM_2.5_ daily average concentration (**b**) the linear regression in Beijing urban area in spring.

Figure 4a illustrates the temporal evolution of daily average concentrations for BC and PM_2.5_ in Beijing during the spring of 2023. The daily average BC concentration stands at 1.39 ± 0.87 μg∙m^−3^, with a concentration range spanning from 0.36 to 3.83 μg∙m^−3^. In comparison, the daily average concentration of PM_2.5_ is recorded at 29.23 ± 29.19 μg∙m^−3^, fluctuating between 2.62 and 141.88 μg∙m^−3^. Overall, the temporal trends in the daily average concentrations of BC and PM_2.5_ exhibit a notable alignment. This coherence is attributed to the characteristic particle size of black carbon, ranging from 0.001 to 1.000 μm, which predominantly adsorbs onto PM_2.5_ particles. The linear analysis (Figure 4b) establishes a statistically positive relationship between the hourly mean concentration of the BC and the PM_2.5_ (r = 0.544), indicating that the trend of their concentration changes over time is basic consistent.

Moreover, this study aggregates BC concentration data from diverse domestic and international cities during the same temporal window (Table 1). In contrast to Lanzhou, where BC concentrations in spring mirror those observed in Beijing, cities such as Xuzhou, Nanjing, Chongqing and Mexico City exhibit springtime average BC concentrations surpassing 3.0 μg∙m^−3^. Notably, in New Delhi, India, characterized by heightened pollution levels, BC concentrations during spring reach a peak of 6.33 μg∙m^−3^. This underscores that, relative to global urban centers, Beijing experiences relatively subdued levels of BC pollution during the spring, underscoring the efficacy of implemented atmospheric pollution control measures.

**Figure 5:**
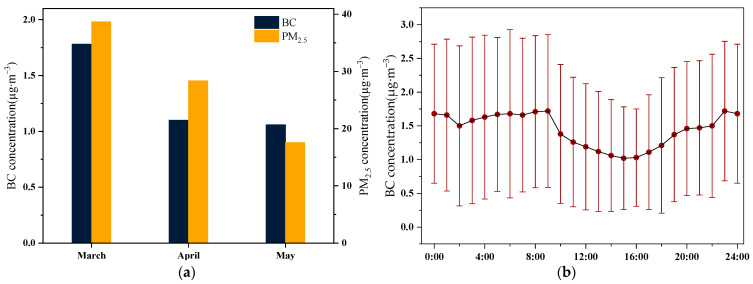
(**a**) Monthly average concentrations of BC and PM_2.5_; (**b**) diurnal variation of BC concentration.

Figure 5a displays the monthly average concentration changes of BC and PM_2.5_ in Beijing’s urban area during the spring of 2023. It is evident that the average concentration of BC and PM_2.5_ was highest in March, while the average concentration of BC remained relatively stable in April and May. This pattern is linked to the prevalence of dust events in Beijing during March, with external dust transport indirectly contributing to elevated PM_2.5_ concentrations. Figure 5b reveals a distinct bimodal structure in the hourly mean BC concentration, consistent with previous research [4,30,31]. The morning peak buildup commences at 6 o’clock, peaks at 9 o’clock and subsequently declines rapidly. This gradual accumulation of BC corresponds to increased traffic volume during the morning commute to work, coupled with the development of surface inversions a few hours after sunrise, leading to the reduced vertical mixing of primary pollutants [32,33]. Moreover, the diurnal variation in BC concentration manifests lower values during the afternoon period from 14:00 to 16:00. This phenomenon is attributable, firstly, to the diurnal fluctuations in boundary layer activity. As solar radiation intensifies over the course of the day, ground temperature gradually ascends, fostering heightened atmospheric turbulence and an elevation in the atmospheric boundary layer. Simultaneously, a marginal increase in wind speed facilitates the vertical transport and dispersion dilution of BC. Secondly, during this timeframe, a discernible reduction in vehicular traffic is observed in comparison to the morning and evening peak hours. In the evening, spanning from 17:00 to 23:00, BC concentrations exhibit a gradual ascent, stabilizing from 23:00 to 05:00 with a slight decline. This shifting trend is ascribed to lower nighttime temperatures, inducing an inversion in atmospheric conditions. Additionally, the influence of evening rush hour traffic contributes to the accumulation of BC within the boundary layer. During the nocturnal period until early morning, marked by diminished human activities, BC undergoes a settling process, resulting in a marginal reduction in its concentration [31,34].

**Figure 6:**
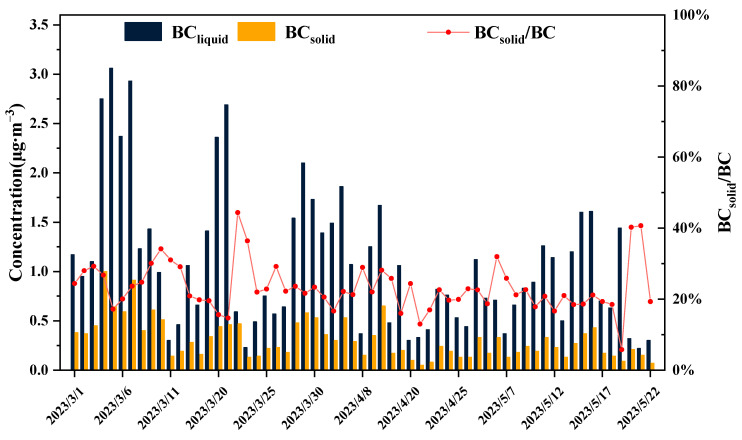
The characteristics of BC concentration by solid fuel and liquid fuel produced, as well as the trend of BC_solid_/BC ratio in spring in Beijing urban area.

In this study, the aethalometer model was employed to estimate the contributions of solid fuel (coal and biomass combustion) and liquid fuel (traffic emissions) to total BC emissions. As depicted in Figure 6, the concentration of BC_liquid_ during spring in Beijing was 1.08 ± 0.71 μg·m^−3^, exceeding that of BC_solid_ (0.31 ± 0.2 μg·m^−3^). The BC_solid_/BC ratio was consistently less than 40%, a finding consistent with the results presented in Figure 3, indicating that liquid fuel combustion is the primary source of black carbon during spring in Beijing. According to the Beijing Municipal Bureau of Statistics (https://tjj.beijing.gov.cn/ (accessed on 30 October 2023)), as of the end of 2022, Beijing had 7.128 million motor vehicles, with less than 10% being new energy vehicles. Diesel and gasoline are the primary liquid fuels, with transportation being their largest consumer [35,36]. This further supports the idea that urban BC emissions primarily originate from liquid fuels. Moreover, the heating season in Beijing has ended, resulting in reduced coal consumption, and significant biomass burning activities that typically occur in spring, such as forest and grassland fires, as well as the burning of uncultivated and cultivated fields, have been rigorously controlled in the surrounding areas of Beijing.

### 3.3. The Relationship between BC and Air Pollutants

**Figure 7:**
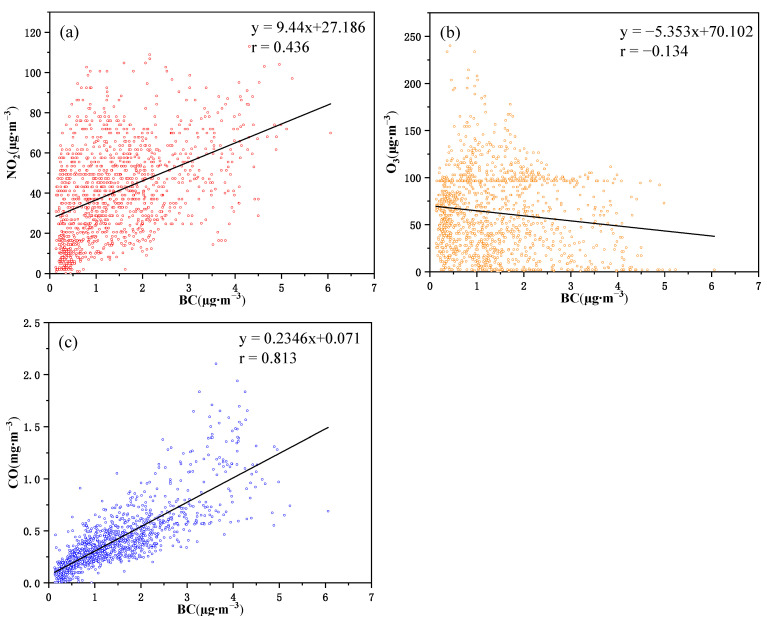
Linear fitting of BC with atmospheric pollutants: (**a**) NO_2_ (Red), (**b**) O_3_ (Orange) and (**c**) CO (Blue).

The sources of black carbon emissions in urban areas are generally stable, primarily stemming from industrial fossil fuel combustion and traffic-related emissions. However, estimating black carbon emissions on a monthly or daily scale presents various challenges and uncertainties, including the determination of emission factors, maintaining emission inventories and the lack of monthly energy consumption data. Therefore, this study employs the correlation between hourly mean concentration of the BC and the atmospheric pollutants (NO_2_, O_3_, CO) to elucidate the potential sources of black carbon emissions in Beijing (Figure 7). The relationship between black carbon mass concentration and other pollutants can, to a certain extent, reflect the characteristics of emission sources. In general, the higher the correlation coefficient, the greater the similarity of emission sources [18,37].

The primary sources of CO in atmospheric pollutants are automobile exhaust emissions, while the primary source of NO_2_ is industrial sources and traffic sources [9,18,38,39]. These two gaseous pollutants also exhibit a positive correlation with black carbon. However, the correlation coefficients for CO (r = 0.812) with black carbon aerosols are larger than that of NO_2_ (r = 0.436), suggesting that automobile exhaust significantly influences the concentration of black carbon in Beijing.

In contrast to other pollutants, O_3_ demonstrates a negative correlation with BC, although this correlation is not statistically significant (r = −0.134). This is due to the fact that O_3_ reacts with NO_x_ in the atmosphere. When NOx concentrations are high, there is greater O_3_ consumption in the chemical reaction. Conversely, when NO_x_ concentrations are low, O_3_ consumption is minimal, resulting in an inverse correlation between the concentration trends of nitrogen oxides and ozone [40]. Therefore, O_3_ is mainly produced by NO_x_ during atmospheric photochemical reactions driven by solar radiation intensity, which differs significantly from the source of BC, leading to a lack of a significant correlation.

### 3.4. Analysis of Potential Pollution Sources Region of BC

#### 3.4.1. Potential Source Regions of BC in Spring

**Figure 8:**
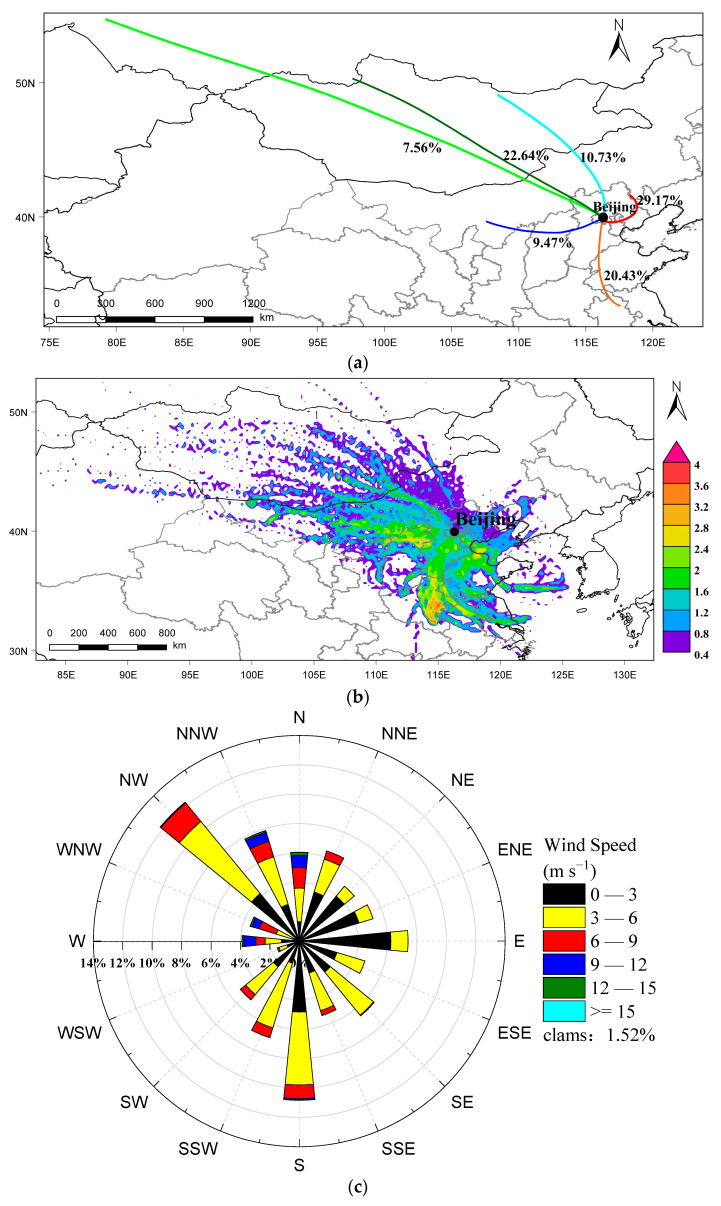
(**a**) Cluster analysis of 48 h backward air mass trajectories arriving at Beijing; (**b**) CWT analysis of the BC; (**c**) wind rose of Beijing for the spring, 2023.

In addition to local source emissions, black carbon emissions from surrounding areas are also significant factors influencing black carbon concentration within Beijing’s urban areas. To assess the impact of regional transport on air pollution in Beijing, we employed the HYSPLIT model to compute air quality trajectory data for Beijing during the spring of 2023. Trajectories were calculated every hour, extending up to 500 m above ground and backtracked for 48 h. Subsequently, we clustered these trajectories (Figure 8a). The analysis revealed that three clusters (7.56%, 22.64%, 10.73%) originated from Mongolia and Siberia in the north and northwest of Beijing: one cluster (9.47%) was traced back to Shanxi and Inner Mongolia to the west of Beijing; another cluster (20.43%) was attributed to Shandong and Anhui in the south of Beijing; and one more cluster (29.17%) was linked to the Beijing–Tianjin–Hebei region near Beijing. This distribution is due to Beijing’s classification under a warm temperate monsoon climate, with the primary spring monsoon wind direction being northwest, followed by south winds (Figure 8c).

Figure 8b presents the CWT analysis results for BC during the spring season in Beijing. The potential source regions for BC during this period are predominantly concentrated in the Beijing–Tianjin–Hebei region, as well as parts of Shanxi, Shandong and Henan provinces. Shanxi provinces, being a pivotal mineral-producing region in China, hosts significant coal and iron ore mines. In contrast, Hebei, Shandong and Henan provinces exhibit high population density, a substantial number of motor vehicles and relatively advanced industrial activities. Furthermore, notably elevated CWT values in the Tianjin and Bohai Bay areas are observed. This phenomenon is attributed to Tianjin’s strategic role as a vital transportation hub in North China, with Tianjin Port ranking as the largest trade port in the northern part of the country. Additionally, the Bohai Bay experiences intensive maritime traffic, where the combustion of liquid fuels from maritime sources significantly contributes to BC concentrations.

#### 3.4.2. Study on the Influence of Spring Dust Weather on BC in Beijing

**Figure 9:**
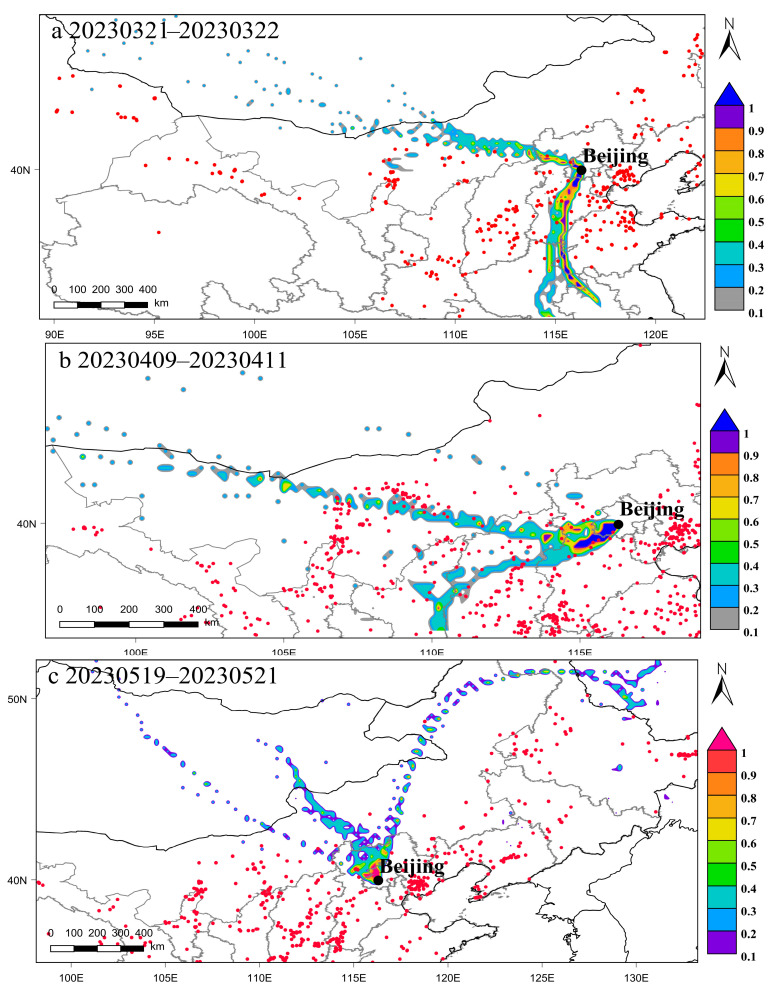
The PSCF analysis during three dust events in spring.

During the spring of 2023, Beijing confronted the implications of recurring dust weather events. This study systematically investigated three representative dust events occurring in the spring season (dust event a: 3.21–3.22; dust event b: 4.9–4.11; dust event c: 5.19–5.21; https://yjglj.beijing.gov.cn/col/col2472/index.html (accessed on 28 November 2023)). Utilizing the PSCF model, an in-depth analysis of the potential source regions of BC associated with these dust events was conducted (Figure 9). Concurrently, a comprehensive examination of concentration trends for BC and PM_10_ during these events was carried out (Figure S1). The primary objective was to elucidate the impact of dust weather on the concentration of BC in Beijing.

Figure 9a elucidates that during dust event a, the predominant potential source areas of BC (PSCF > 0.7) are predominantly situated in the southern regions of Beijing, encompassing Hebei and Henan province. Conversely, contributions from the northern and northwestern directions of Beijing manifest negligible levels of black carbon. Insights gleaned from the research of Chen et al. [41] suggest that the primary sources of spring dust in northern China emanate from the deserts of Mongolia and the Taklamakan in Xinjiang. Furthermore, observations derived from Figure S1a illustrate that as PM_10_ concentration experiences a rapid increase, BC concentration undergoes a gradual descent.

In dust event b (Figure 9b), the principal potential source areas of BC are identified in the southwestern direction of Beijing, encompassing regions such as Hebei Baoding and Shanxi Datong. Interestingly, this direction does not align with the primary sources of dust, and the concentration trends of PM10 and BC are notably consistent with those observed in dust event a. Concerning dust event c (Figure 9c), the principal potential source areas of BC are concentrated within the Beijing area. Notably, dust event c encompasses two distinct dust processes (Figure S1c). During both of these episodes, PM_10_ concentrations exhibit marked variability, while BC concentrations tend to remain stable, with a marginal increase during the intervals separating the two dust processes.

In conclusion, contrary to expectations, springtime dust event does not result in an escalation of BC concentrations in Beijing. Instead, the heightened wind speeds during dust events contribute to an accelerated dispersion of pollutants.

## 4. Conclusions

Utilizing data obtained from the multi-wavelength aethalometer, coupled with meteorological and atmospheric pollutant datasets, our study provides a comprehensive investigation into the optical absorption properties, concentration dynamics and potential sources of BC in Beijing during the spring of 2023. Our findings reveal that both Abs_BC_ and Abs_BrC_ exhibit maximal absorption at 370 nm, diminishing with increasing wavelength. The values for Abs_(880nm)BC_ and Abs_(370nm)BrC_ are reported as 10.82 ± 6.8 Mm^−1^ and 11.471 ± 10.87 Mm^−1^, respectively, with Abs_(370nm)BrC_ accounting for less than 40% throughout the study period. The average concentration of BC during spring is documented as 1.39 ± 0.87 μg∙m^−3^, concurrently with PM_2.5_ registering an average concentration of 29.23 ± 29.19 μg∙m^−3^, both reaching their zenith in March. Furthermore, their concentration trends exhibit a basic consistency, establishing a positive correlation (r = 0.544). The diurnal variability of BC in Beijing manifests as a distinctive bimodal structure. Aethalometer model outcomes suggest that BC concentration from liquid fuel combustion (1.08 ± 0.71 μg∙m^−3^) significantly surpasses that from solid fuel combustion (0.31 ± 0.2 μg∙m^−3^). BC demonstrates a substantial positive correlation with NO_2_ (r = 0.436) and CO (r = 0.813), while its correlation with O_3_ (r = −0.134) lacks statistical significance, underscoring liquid fuel combustion as the predominant source of BC in Beijing. Trajectory cluster analysis reveals that the primary air mass trajectories in Beijing originate from the northwest. Simultaneously, CWT analysis highlights that external sources of BC in Beijing during spring predominantly concentrate in neighboring cities and regions, as well as parts of the Henan, Shandong and Shanxi provinces. Importantly, dust processes do not engender drastic fluctuations in BC concentrations in Beijing. This study contributes pivotal data on the daily average concentration, optical characteristics and source distribution of BC in Beijing during the spring, thereby laying a solid foundation for climate change simulations, public health impact assessments and studies pertaining to relative emission reductions.

## Figures and Tables

**Table 1:** Comparison of BC concentrations in various local regions.

Place	Observation Period	BC Concentration (μg∙m^−3^)	Study
Beijing, China	2 March 2023 to 22 May 2023	1.39 ± 0.87	This study
Xuzhou, China	Spring, 2014	3.308	[4]
Nanjing, China	Spring, 2018	3.351 ± 0.919	[27]
Chongqin, China	17 January 2020 to 1 April 2020	3.8 ± 3.0	[28]
Lanzhou, China	Spring, 2019	1.42 ± 0.63	[22]
New Delhi, India	April to June 2016	6.33	[29]
Mexico City, Mexico	April to June 2016	3.22 ± 1.54	[9]

## Data Availability

The data presented in this study are available on request from the corresponding author.

## Supplementary Materials

The following supporting information can be downloaded at: https://www.mdpi.com/article/10.3390/toxics12030202/s1, Figure S1. The Mean hourly concentration trend of BC and PM_10_ in the three dust events (a) 0:00, 21 March–23:00 22 March; (b) 12:00, 9 April–13:00, 11 April; (c) 19:00, 19 May–7:00, 22 May.

## References

[1] Lelieveld J., Evans J.S., Fnais M., Giannadaki D., Pozzer A. (2015). The contribution of outdoor air pollution sources to premature mortality on a global scale. Nature.

[2] Lave L.B., Seskin E.P. (2013). Air Pollution and Human Health.

[3] Bond T.C., Doherty S.J., Fahey D.W., Forster P.M., Berntsen T., DeAngelo B.J., Flanner M.G., Ghan S., Kärcher B., Koch D. (2013). Bounding the role of black carbon in the climate system: A scientific assessment. J. Geophys. Res.-Atmos..

[4] Chen W., Tian H.M., Zhao H.M., Qin K. (2020). Multichannel characteristics of absorbing aerosols in Xuzhou and implication of black carbon. Sci. Total Environ..

[5] Laeremans M., Dons E., Avila-Palencia I., Carrasco-Turigas G., Orjuela-Mendoza J.P., Anaya-Boig E., Cole-Hunter T., De Nazelle A., Nieuwenhuijsen M., Standaert A. (2018). Black Carbon Reduces the Beneficial Effect of Physical Activity on Lung Function. Med. Sci. Sport. Exer..

[6] Lai C.H., Lin C.H., Liao C.C. (2017). Respiratory deposition and health risk of inhalation of particle-bound heavy metals in the carbon black feeding area of a tire manufacturer. Air Qual. Atmos. Health.

[7] Louwies T., Nawrot T., Cox B., Dons E., Penders J., Provost E., Panis L.I., De Boever P. (2015). Blood pressure changes in association with black carbon exposure in a panel of healthy adults are independent of retinal microcirculation. Environ. Int..

[8] Huang R.J., Yang L., Cao J.J., Chen Y., Chen Q., Li Y.J., Duan J., Zhu C.S., Dai W.T., Wang K. (2018). Brown Carbon Aerosol in Urban Xi’an, Northwest China: The Composition and Light Absorption Properties. Environ. Sci. Technol..

[9] Peralta O., Ortínez-Alvarez A., Basaldud R., Santiago N., Alvarez-Ospina H., de la Cruz K., Barrera V., Espinosa M.D., Saavedra I., Castro T. (2019). Atmospheric black carbon concentrations in Mexico. Atmos. Res..

[10] Barman N., Gokhale S. (2019). Urban black carbon-source apportionment, emissions and long-range transport over the Brahmaputra River Valley. Sci. Total Environ..

[11] Li X.R., Sun N.N., Jin Q.H., Zhao Z.Y., Wang L.L., Wang Q.L., Gu X., Li Y.X., Liu X.A. (2022). Light absorption properties of black and brown carbon in winter over the North China Plain: Impacts of regional biomass burning. Atmos. Environ..

[12] Lou S.J., Yang Y., Wang H.L., Smith S.J., Qian Y., Rasch P.J. (2019). Black Carbon Amplifies Haze Over the North China Plain by Weakening the East Asian Winter Monsoon. Geophys. Res. Lett..

[13] Liu Q.Y., Ma T.M., Olson M.R., Liu Y.J., Zhang T.T., Wu Y., Schauer J.J. (2016). Temporal variations of black carbon during haze and non-haze days in Beijing. Sci. Rep..

[14] Wang H.Q., He Q.S., Chen Y.H., Kang Y.M. (2014). Characterization of black carbon concentrations of haze with different intensities in Shanghai by a three-year field measurement. Atmos. Environ..

[15] Huang G., Liu W., Liu Z.H., Zhang Y. (2015). A Research Overview of Black Carbon Aerosols. J. Catastrophology.

[16] Yang Y., Wang H.L., Smith S.J., Ma P.L., Rasch P.J. (2017). Source attribution of black carbon and its direct radiative forcing in China. Atmos. Chem. Phys..

[17] Liu Y., Yan C.Q., Zheng M. (2018). Source apportionment of black carbon during winter in Beijing. Sci. Total Environ..

[18] Jing A.K., Zhu B., Wang H.L., Yu X.N., An J.L., Kang H. (2019). Source apportionment of black carbon in different seasons in the northern suburb of Nanjing, China. Atmos. Environ..

[19] Yang Y., Zhao D.L., Huang Y., Tian P., Liu D.T., Huang M.Y., He H., Ding D.P., Li Y.Y., Zhao C. (2022). Effects of black carbon aerosol on air quality and vertical meteorological factors in early summer in Beijing. Sci. Total Environ..

[20] Zhang Q., Streets D.G., Carmichael G.R., He K.B., Huo H., Kannari A., Klimont Z., Park I.S., Reddy S., Fu J.S. (2009). Asian emissions in 2006 for the NASA INTEX-B mission. Atmos. Chem. Phys..

[21] Hsu Y.K., Holsen T.M., Hopke P.K. (2003). Comparison of hybrid receptor models to locate PCB sources in Chicago. Atmos. Environ..

[22] Chen P.F., Kang S.C., Gan Q.Y., Yu Y., Yuan X.L., Liu Y.J., Tripathee L., Wang X.X., Li C.L. (2023). Concentrations and light absorption properties of PM2.5 organic and black carbon based on online measurements in Lanzhou, China. J. Environ. Sci..

[23] Liakakou E., Stavroulas I., Kaskaoutis D.G., Grivas G., Paraskevopoulou D., Dumka U.C., Tsagkaraki M., Bougiatioti A., Oikonomou K., Sciare J. (2020). Long-term variability, source apportionment and spectral properties of black carbon at an urban background site in Athens, Greece. Atmos. Environ..

[24] Xie C.H., Xu W.Q., Wang J.F., Wang Q.Q., Liu D.T., Tang G.Q., Chen P., Du W., Zhao J., Zhang Y.J. (2019). Vertical characterization of aerosol optical properties and brown carbon in winter in urban Beijing, China. Atmos. Chem. Phys..

[25] Lack D.A., Langridge J.M., Bahreini R., Cappa C.D., Middlebrook A.M., Schwarz J.P. (2012). Brown carbon and internal mixing in biomass burning particles. Proc. Natl. Acad. Sci. USA.

[26] Li S., Zhu M., Yang W.Q., Tang M.J., Huang X.L., Yu Y.G., Fang H., Yu X., Yu Q.Q., Fu X.X. (2018). Filter-based measurement of light absorption by brown carbon in PM in a megacity in South China. Sci. Total Environ..

[27] Yang X.-M., Shi S.-S., Zhang C., Wang H.-L., Wang Z.-B., Zhu B. (2020). Temporal Evolution and Main Influencing Factors of Black Carbon Aerosol in Nanjing. Environ. Sci..

[28] Chen Y., Zhang S.M., Peng C., Shi G.M., Tian M., Huang R.J., Guo D.M., Wang H.B., Yao X.J., Yang F.M. (2020). Impact of the COVID-19 pandemic and control measures on air quality and aerosol light absorption in Southwestern China. Sci. Total Environ..

[29] Sharma M.C., Pandey V.K., Kumar R., Latief S.U., Chakrawarthy E., Acharya P. (2018). Seasonal characteristics of black carbon aerosol mass concentrations and influence of meteorology, New Delhi (India). Urban. Clim..

[30] Kumar R.R., Soni V.K., Jain M.K. (2020). Evaluation of spatial and temporal heterogeneity of black carbon aerosol mass concentration over India using three year measurements from IMD BC observation network. Sci. Total Environ..

[31] Chen W., Tian H.M., Qin K. (2019). Black Carbon Aerosol in the Industrial City of Xuzhou, China: Temporal Characteristics and Source Appointment. Aerosol Air Qual. Res..

[32] Williams M.A., Kumar T.V.L., Rao D.N. (2019). Characterizing black carbon aerosols in relation to atmospheric boundary layer height during wet removal processes over a semi urban location. J. Atmos. Sol.-Terr. Phys..

[33] Xiao S.H., Yu X.N., Zhu B., Kumar K.R., Li M., Li L. (2020). Characterization and source apportionment of black carbon aerosol in the Nanjing Jiangbei New Area based on two years of measurements from Aethalometer. J. Aerosol Sci..

[34] Bibi S., Alam K., Chishtie F., Bibi H., Rahman S. (2017). Temporal variation of Black Carbon concentration using Aethalometer observations and its relationships with meteorological variables in Karachi, Pakistan. J. Atmos. Sol.-Terr. Phys..

[35] Fang D., Yang J. (2021). Drivers and critical supply chain paths of black carbon emission: A structural path decomposition. J. Environ. Manag..

[36] Song W.W., He K.B., Lei Y. (2012). Black carbon emissions from on-road vehicles in China, 1990–2030. Atmos. Environ..

[37] Wang Y.X., Wang X., Kondo Y., Kajino M., Munger J.W., Hao J.M. (2011). Black carbon and its correlation with trace gases at a rural site in Beijing: Top-down constraints from ambient measurements on bottom-up emissions. J. Geophys. Res.-Atmos..

[38] Streets D.G., Yarber K.F., Woo J.H., Carmichael G.R. (2003). Biomass burning in Asia: Annual and seasonal estimates and atmospheric emissions. Glob. Biogeochem. Cycles.

[39] Duan F.K., He K.B., Ma Y.L., Jia Y.T., Yang F.M., Lei Y., Tanaka S., Okuta T. (2005). Characteristics of carbonaceous aerosols in Beijing, China. Chemosphere.

[40] Tan Y., Wang H.L., Zhu B., Zhao T.L., Shi S.S., Liu A.K., Liu D.Y., Pan C., Cao L. (2022). The interaction between black carbon and planetary boundary layer in the Yangtze River Delta from 2015 to 2020: Why O3 didn’t decline so significantly as PM_2.5_. Environ. Res..

[41] Chen S., Zhao D., Huang J., He J., Chen Y., Chen J., Bi H., Lou G., Du S., Zhang Y. (2023). Mongolia Contributed More than 42% of the Dust Concentrations in Northern China in March and April 2023. Adv. Atmos. Sci..

