# Supplementary material for: Pollution Characteristics and Source Apportionment of Black Carbon Aerosols during Spring in Beijing
Source: Toxics. 2024 Mar 5;12(3):202. doi: 10.3390/toxics12030202 (PMC10975136; doi:10.3390/toxics12030202)
Supplement: Supplementary file 1 [file toxics-12-00202-s001.zip › toxics-2868789-supplementary.pdf]

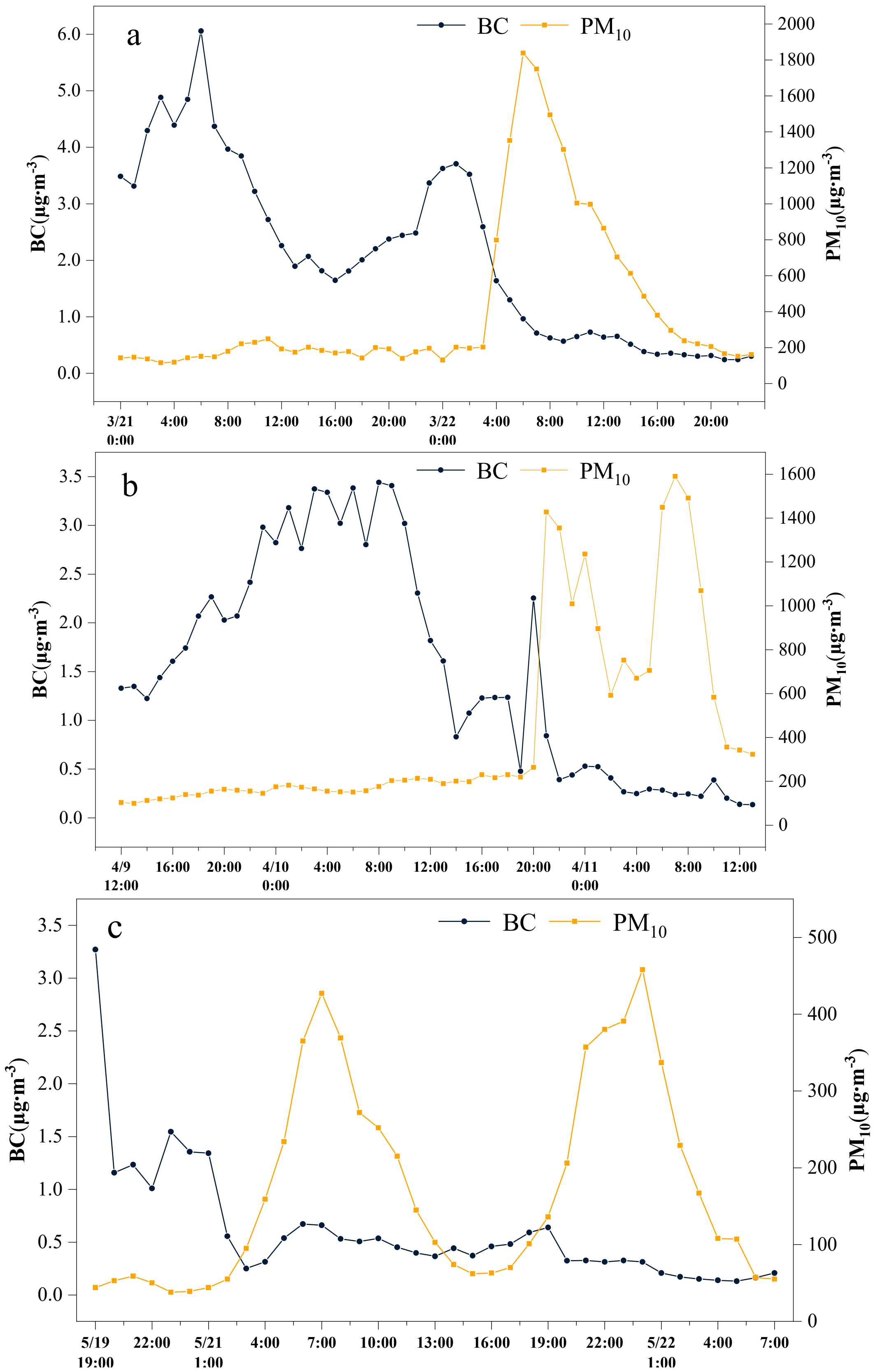

Figure S1. The Mean hourly concentration trend of BC and PM_{10} in the three dust events (a) 0:00, 21 March—23:00 22 March; (b) 12:00, 9 April—13:00, 11 April; (c) 19:00, 19 May—7:00, 22 May.
